# Visualization of the Anterior Temporal Artery as a Predictor of Outcome in Middle Cerebral Artery Occlusion Patients Achieving Successful Recanalization After Transfer

**DOI:** 10.7759/cureus.25173

**Published:** 2022-05-20

**Authors:** Jonathan M Parish, Jeremy B Rhoten, Dale Strong, Tanushree Prasad, Andrew Hines, Joe D Bernard, Jonathan Clemente, Rahul Karamchandani, Andrew W Asimos, William R Stetler

**Affiliations:** 1 Neurological Surgery, Carolina Neurosurgery & Spine Associates, Charlotte, USA; 2 Neurological Surgery, Carolinas Medical Center, Charlotte, USA; 3 Neurology, Atrium Health, Charlotte, USA; 4 Information and Analytics Services, Atrium Health, Charlotte, USA; 5 Interventional Radiology, Charlotte Radiology, Charlotte, USA; 6 Radiology, Charlotte Radiology, Charlotte, USA; 7 Emergency Medicine, Atrium Health, Charlotte, USA

**Keywords:** cerebral perfusion imaging, neuroimaging, computed tomography angiography, thrombectomy, stroke

## Abstract

Introduction

Anterior temporal artery (ATA) visualization on computed tomography angiography (CTA) has been previously associated with good outcomes in middle cerebral artery (MCA) occlusions, but not in the setting of patients who initially present to non-thrombectomy centers.

Methods

We retrospectively identified acute MCA (M1) occlusion patients who underwent mechanical thrombectomy after transfer from non-thrombectomy-capable centers. Neuroradiologists confirmed the MCA (M1) as the most proximal site of occlusion on CTA and assessed for visualization of the ATA. Thrombolysis in Cerebral Infarction (TICI) 2b or greater revascularization scores were confirmed by neurointerventionalists blinded to patient outcomes. Ninety-day modified Rankin scale (mRS) scores were obtained via a structured telephone questionnaire.

Results

We identified 102 M1 occlusion patients over a three-and-a-half-year period presenting to a non-thrombectomy-capable center who underwent transfer and mechanical thrombectomy. There were no significant differences in age, gender, race, comorbidities, or median National Institute of Health Stroke Scale (NIHSS) scores between the ATA visualized (n = 47) versus non-visualized (n = 55) cohort, and no significant differences in baseline Alberta Stroke Program Early Computed Tomography (ASPECT) scores, post-intervention TICI scores, or interval from last known well to revascularization. There was a strong trend in functional independent outcome (mRS ≤ 2) for patients with ATA visualization (63.8% vs. 45.5%, p = 0.064).

Conclusion

For patients presenting to non-thrombectomy centers without CT perfusion capability, ATA visualization should be further investigated as an outcome predictor, given its association with functional independence after successful recanalization.

This article was previously presented as a meeting abstract at the 2021 International Stroke Conference on March 17-19, 2021.

## Introduction

In acute ischemic stroke, time from symptom onset to revascularization and revascularization quality are known to be predictors of outcome [1-3]. Computed tomography perfusion (CTP) imaging has become part of the routine evaluation of acute ischemic stroke patients with suspected large vessel occlusion. In particular, for patients presenting between six and 24 hours from symptom onset, CTP is used to select candidates for endovascular thrombectomy (EVT) [4,5]. Nonetheless, despite the expanded use of CTP technology, non-diagnostic studies sometimes occur as a result of head motion, a failed bolus injection, poor scan timing, and low signal-to-noise [6]. Moreover, not all patients present to centers with CTP capability. Therefore, developing non-CTP-dependent radiographic criteria to predict the response to EVT would be beneficial.

The anterior temporal artery (ATA) is commonly the most proximal major branch of the middle cerebral artery (MCA), identified in over 80% of cadaveric studies [7]. Visualization of the ATA on computed tomography angiography (CTA) has been found to be a predictor of good outcomes and lower mortality rate in MCA occlusions [8,9]. Correspondingly, studies have shown more distal MCA occlusions that maintain patency of the proximal lenticulostriate vessels result in improved disability-free outcomes [10]. This is likely due to important collateral flow, as well as preservation of blood supply to the basal ganglia. However, ATA visualization as a predictor of outcome, specifically in the setting of successful mechanical thrombectomy, has never been reported. Consequently, the aim of this study was to evaluate whether the presence of the ATA on CTA source images in patients with proximal MCA (M1) occlusions predicts outcomes in patients successfully treated with EVT who initially present to non-thrombectomy-capable centers.

## Materials and methods

We screened our healthcare system’s prospectively maintained stroke database from January 1, 2017, to May 15, 2020, for all patients requiring transfer for EVT involving target occlusions of the MCA after obtaining IRB approval from Carolinas Medical Center IRB (03-21-21E). Our institutional guidelines for EVT are listed in Table 1.

**Table 1:** Institutional guidelines for endovascular thrombectomy candidacy NIHSS: National Institute of Health Stroke Scale; mRS: modified Rankin scale; ICA: internal carotid artery; M1: middle cerebral artery M1 branch; M2: middle cerebral artery M2 branch; ASPECTS: Alberta Stroke Program Early Computed Tomography Score; Core infarct: cerebral blood flow reduction to <30% of the corresponding contralateral territory.

Time window	NIHSS	Baseline mRS	Site of occlusion	ASPECTS	Perfusion imaging
0-6 hours	≥6 or disabling deficit	mRS 0-2	ICA, M1, proximal M2	ASPECTS ≥ 6	N/A
6-16 hours	≥6 or disabling deficit	mRS 0-2	ICA, M1, proximal M2	ASPECTS ≥ 6	Core infarct < 70 cc, mismatch volume ≥ 15 cc, and mismatch ratio ≥ 1.8
16-24 hours	≥10	mRS 0-2	ICA, M1, proximal M2	ASPECTS ≥ 6	If ≥ 80 yo, core < 21 cc; if < 80 yo: NIHSS ≥ 10, core < 31 cc or NIHSS ≥ 20 and core 31-50 cc

**Figure 1:**
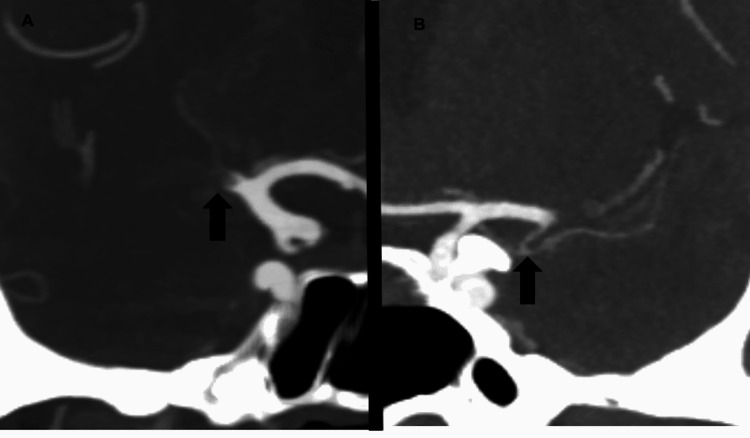
Coronal CT angiography example of the presence and absence of the anterior temporal artery (A) Right M1 proximal occlusion with no visualization of the anterior temporal artery. (B) Left M1 occlusion with patent anterior temporal artery.

For all patients identified on our initial screen, one of three board-certified neuroradiologists blinded to patient outcomes confirmed the MCA M1 as the most proximal site of occlusion on CTA and assessed for visualization of ATA (Figures 1A, 1B).

One of four interventional neuroradiologists or neurosurgeons blinded to patient outcomes reviewed the post-EVT digital subtraction angiogram (DSA) to assign a Thrombolysis in Cerebral Infarction (TICI) scale score [11]. For purposes of our analysis, DSAs meeting 2b and 2c criteria were grouped together. The interpreter assigning the TICI score was not the interventionalist who performed the thrombectomy and rendered the initial TICI score. Any discrepancies between TICI scores assigned blindly and the TICI scores initially assigned by the neurointerventionalist performing the procedure were adjudicated by a board-certified neuroradiologist. For our final analysis, we excluded all patients with a revascularization TICI score less than 2b. Demographic and clinical data were collected by trained data abstractors using explicitly defined criteria, based on established standard operating procedures for our code stroke patient database. Our outcome measurement was a modified Rankin scale (mRS) score 90 days after EVT, obtained via telephone utilizing a structured questionnaire [12]. We defined independent outcome as an mRS score of 0-2.

Since we have deployed CTP at many of the non-interventional sites within our integrated stroke network, we additionally explored the relationship of ATA visualization to corresponding CTP parameters. Estimates of the volume of the ischemic core and penumbral regions from CTP were calculated with the use of iSchemaView RAPID software (San Mateo, CA). The ischemic core lesion was defined as a cerebral blood flow (CBF) reduction to <30% of the corresponding contralateral territory and the total hypoperfused volume was defined as the volume of tissue with Tmax > six seconds.

Statistical analyses

Descriptive statistics, including median and interquartile ranges for continuous variables, or counts and percentages for categorical variables, were used to describe the study sample. We made comparisons between groups using Wilcoxon two-sample tests for continuous variables, and chi-square or Fisher’s exact tests for categorical variables. The level of significance was set at p < 0.05 for all comparisons. We analyzed data using standard statistical methods utilizing SAS 9.4 (SAS Institute Inc., Cary, NC).

## Results

We identified a total of 102 MCA M1 occlusion patients over a three-and-a-half-year period presenting to non-thrombectomy-capable centers (Table 2).

**Table 2:** Characteristics of patients undergoing mechanical thrombectomy after presenting to non-thrombectomy-capable centers * Mismatch volume = difference in volume between total hypoperfused area and core infarct (Tmax > 6 s volume minus CBF < 30 volume). IQR: interquartile range; NIHSS: National Institute of Health Stroke Scale; ASPECT: Alberta Stroke Program Early Computed Tomography; TICI: Thrombolysis in Cerebral Infarction; ATA: anterior temporal artery; IA: intra-arterial thrombectomy; IV tPA: intravenous tissue plasminogen activator; LKW: last known well; CBF: cerebral blood flow (core infarct); Tmax: time to maximum (hypoperfused area).

Characteristics	ATA not visualized (n = 55)	ATA visualized (n = 47)	P-value
Demographic			
Age, years, median (IQR)	72 (58-83)	68 (59-79)	0.358
Sex, male, n (%)	27 (49.1)	19 (40.4)	0.381
Race, n (%)			
Caucasian	39 (70.9)	31 (65.9)	0.762
African American	11 (20.0)	13 (27.7)	
All others	5 (9.1)	3 (6.4)	
Ethnicity, n (%)			
Hispanic or Latino	4 (7.3)	2 (4.3)	0.871
Non-Hispanic or Latino	49 (89.1)	44 (93.6)	
Unknown	2 (3.6)	1 (2.1)	
Comorbidities, n (%)			
Hypertension	38 (69.1)	38 (80.9)	0.174
Hyperlipidemia	24 (43.6)	24 (51.1)	0.454
Diabetes	11 (20.0)	15 (31.9)	0.169
Atrial fibrillation	24 (43.6)	14 (29.8)	0.149
Smoking	18 (32.7)	15 (31.9)	0.930
Baseline NIHSS, median (IQR)	17 (13-22)	16 (10-21)	0.397
Wake-up stroke, n (%)	9 (16.7)	16 (34.8)	0.037
Patient classification, n (%)			
IA only	30 (54.6)	25 (53.2)	0.891
IV tPA + IA	25 (45.4)	22 (46.8)	
LKW to final revascularization time interval (min), median (IQR)	362 (220-502)	420 (247-829)	0.095
Imaging			
M1 right, n (%)	27 (49.1)	13 (27.7)	0.027
ASPECT score, median (IQR)	10 (9-10)	10 (9-10)	0.553
CBF < 30% volume, cc, median (IQR)	8 (0-40)	0 (0-7)	0.001
Tmax > 6 s volume, cc, median (IQR)	125 (101-201)	107 (69-139)	0.004
Mismatch volume*, cc, median (IQR)	122 (81-158)	101 (66-125)	0.073
Post-TICI score			
2b	35 (63.6)	28 (59.6)	0.674
3	20 (36.4)	19 (40.4)	

**Figure 2:**
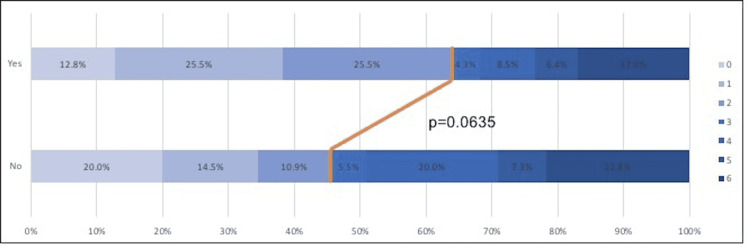
Ninety-day modified Rankin scale (mRS) outcomes for all patients undergoing mechanical thrombectomy after presenting to non-thrombectomy-capable centers

There were no significant differences in age, gender, race, comorbidities, or median National Institute of Health Stroke Scale (NIHSS) scores between the ATA visualized (n = 47) versus the not visualized (n = 55) cohort, but there were significantly more wake-up strokes in the ATA visualized group (34.8% vs. 16.7%, p = 0.0371). With respect to imaging variables, as demonstrated in Table 2, there were no significant differences in baseline Alberta Stroke Program Early Computed Tomography (ASPECT) scores or post-intervention TICI scores (p = 0.5532 and p = 0.6739, respectively). However, the estimated ischemic core infarct volumes, based on the CBF < 30% volumes on CTP, were significantly lower in the ATA visualized group. Also, there were significantly more right M1 occlusions in patients in which the ATA was not visualized (p = 0.0271). There was a nonsignificant strong trend for achieving independent outcomes at 90 days after EVT for patients with ATA visualization, compared to those for whom the ATA was not visualized on the CTA (63.8% vs. 45.5%, p = 0.064) (Figure 2).

Based on this trend, we analyzed for additional independent predictors of good (mRS <= 2) versus poor outcome (mRS > 2) (Table 3).

**Table 3:** Independent predictors of independent outcomes for patients undergoing mechanical thrombectomy after presenting to non-thrombectomy-capable centers * Mismatch volume = difference in volume between total hypoperfused area and core infarct (Tmax > 6 s volume minus CBF < 30 volume) mRS: modified Rankin Scale; NIHSS: National Institute of Health Stroke Scale; IA: intra-arterial thrombectomy; IV tPA: intravenous tissue plasminogen activator; ASPECT: Alberta Stroke Program Early Computed Tomography; CBF: cerebral blood flow (core infarct); Tmax: time to maximum (hypoperfused area); ATA: anterior temporal artery; LKW: last known well; CTA: computed tomography angiography.

	mRS > 2 (n = 47)	mRS ≤ 2 (n = 55)	P-value
Demographic			
Age, years, median (IQR)	78 (63-87)	65 (57-76)	0.0003
Sex, male, n (%)	19 (40.4)	27 (49.1)	0.381
Race, n (%)			
Caucasian	31 (65.9)	30 (70.9)	0.319
African American	10 (21.3)	14 (25.5)	
All others	6 (12.8)	2 (3.6)	
Ethnicity, n (%)			
Hispanic or Latino	4 (8.5)	2 (3.6)	0.475
Non-Hispanic or Latino	41 (87.2)	52 (94.6)	
Unknown	2 (4.3)	1 (1.8)	
Comorbidities, n (%)			
Hypertension	39 (83)	37 (67.3)	0.07
Hyperlipidemia	25 (53.2)	23 (41.8)	0.251
Diabetes	11 (23.4)	15 (27.3)	0.655
Atrial fibrillation	21 (44.7)	17 (30.9)	0.152
Smoking	15 (31.9)	18 (32.7)	0.93
Baseline NIHSS, median (IQR)	20 (15-24)	14 (9-18)	< 0.0001
Wake-up stroke, n (%)	7 (15.6)	18 (32.7)	0.05
Patient classification, n (%)			
IA only	28 (59.6)	27 (49.1)	0.29
IV tPA + IA	19 (40.4)	28 (50.9)	
Imaging			
M1 right, n (%)	15 (31.9)	25 (45.5)	0.163
ASPECT score, median (IQR)	10 (9.5-10)	10 (9-10)	0.43
CBF < 30% volume, cc, median (IQR)	6 (0-21)	0 (0-18)	0.355
Tmax > 6 s volume, cc, median (IQR)	124 (89-159)	110 (83-150)	0.311
ATA visualized, n (%)	17 (36.2)	30 (54.5)	0.064
Process			
LKW to final revascularization time interval (min), median (IQR)	420 (252-661)	321 (230-502)	0.272
CTA acquisition to groin puncture time interval (min), median (IQR)	110 (92-140)	109 (86-120)	0.136
CTA acquisition to first pass time interval (min), median (IQR)	135 (111-157)	122 (108-140)	0.085

Younger age, lower baseline NIHSS, and wake-up stroke were significantly associated with independent outcomes. The visualization of the ATA was nearest to the clinical significance of the remaining variables including perfusion and process variables.

## Discussion

Since many non-thrombectomy centers lack CTP capability, there is a need for imaging outcome predictors derived from the non-contrast computed tomography (NCCT) head or a single-phase CTA. ASPECTS scoring from the NCCT head [13] can be utilized but its high interobserver variability undermines the practicality of its use [14,15]. Additionally, while CTA-based collateral scoring can be utilized [9,10], the scores that perform best are complex [16] and are likely considerably more challenging to derive than a binary interpretation of the absence or presence of ATA visualization. Our data strongly suggest that assessing for visualization of the ATA in patients with acute MCA M1 occlusions is predictive of the outcome if successful revascularization can be achieved after transfer for thrombectomy. While missing statistical significance, there was an 18% difference in independent function for patients presenting initially to non-thrombectomy-capable centers for whom the ATA was visualized versus not visualized. Furthermore, it is possible that as with collateral grading, combining ATA visualization with outcome prediction scores will result in a better prediction of poor outcomes after transfer for EVT compared to the prediction scores alone [17].

An additional finding of our analysis, which is consistent with other predictive models, is that age and NIHSS were significantly associated with favorable outcomes [18,19]. Interestingly, in patients presenting to non-thrombectomy centers, last known well to revascularization and perfusion variables were not significantly associated with independent outcomes. Also notable is that patients for whom the ATA was not visualized were significantly more likely to have occlusions involving the right versus the left M1. Since left M1 occlusions are more likely to be functionally disabling due to the aphasia they often produce, the imbalance in hemispheric involvement of our study cohort is unlikely to explain our findings.

It is understandable in our study population, which included only patients who underwent thrombectomy, that there were more wake-up strokes in the ATA visualized patient group. Because ATA visualization is likely associated with patency of the proximal lenticulostriate vessels and preserved blood supply to the basal ganglia, it is plausible that such patients in a wake-up cohort would be likely to have more favorable perfusion profiles thereby qualifying for EVT. Indeed, the differences in perfusion parameters between the ATA visualized and non-visualized group bears this out. ATA visualization was significantly associated with lower overall ischemic core and hypoperfused volumes. Since we found no association with ASPECTS scores between groups, our results indicate ATA visualization adds predictive value that cannot be obtained from NCCT alone.

Our study has some limitations, including a retrospective design, but it should be emphasized that while all cases were initially identified from a query of our large stroke network database, all CTA images were reinterpreted by neuroradiologists to verify that the MCA was the most proximal site of vessel occlusion and to assess for visualization of the ATA. The ability of non-board certified neuroradiology readers to routinely identify the absence or presence of the ATA was not evaluated as part of our analysis. As discussed earlier, the ATA is the first major branch in >80% of cadaveric studies and the lack of visualization does not necessarily correlate with a more proximal occlusion. Additionally, since it has already been reported that even among interventional and diagnostic neuroradiologists there is insufficient agreement in ASPECTS scoring [15], we relied on the ASPECTS score assigned by the neuroradiologist who initially interpreted the NCCT study. However, all TICI scores initially assigned by the interventionist were blindly reviewed and adjudicated by a third reader when required. Also, a priori to conducting our analysis, we decided to combine TICI 2b and TICI 2c scores in one category, though there is literature supporting improved outcomes in patients with 2c versus 2b scores. A final limitation is our inclusion of only patients selected to undergo EVT.

## Conclusions

For MCA occlusion patients initially presenting to non-thrombectomy centers achieving successful recanalization, there is a strong trend for visualization of the ATA being a predictor of independent functional outcome. Current common thrombectomy selection criteria of last known well time to revascularization and favorable perfusion imaging variables were not significantly associated with outcome and encourage further studies to determine thrombectomy selection criteria for patients presenting to non-thrombectomy centers. Especially for institutions without CTP capability, this association with ATA visualization should be further investigated as a predictor of good outcomes after transfer for successful mechanical thrombectomy.
